# Comparative route of administration studies using therapeutic siRNAs show widespread gene modulation in Dorset sheep

**DOI:** 10.1172/jci.insight.152203

**Published:** 2021-12-22

**Authors:** Chantal M. Ferguson, Bruno M.D.C. Godinho, Julia F. Alterman, Andrew H. Coles, Matthew Hassler, Dimas Echeverria, James W. Gilbert, Emily G. Knox, Jillian Caiazzi, Reka A. Haraszti, Robert M. King, Toloo Taghian, Ajit Puri, Richard P. Moser, Matthew J. Gounis, Neil Aronin, Heather Gray-Edwards, Anastasia Khvorova

**Affiliations:** 1 RNA Therapeutics Institute and; 2 Department of Radiology, New England Center for Stroke Research, University of Massachusetts Medical School, Worcester, Massachusetts, USA.; 3 Department of Biomedical Engineering, Worcester Polytechnic Institute, Worcester, Massachusetts, USA.; 4 Neurointerventional Radiology,; 5 Department of Neurosurgery, and; 6 Department of Molecular Medicine, University of Massachusetts Medical School, Worcester, Massachusetts, USA.

**Keywords:** Neuroscience, Gene therapy, Neurodegeneration, RNA processing

## Abstract

siRNAs comprise a class of drugs that can be programmed to silence any target gene. Chemical engineering efforts resulted in development of divalent siRNAs (di-siRNAs), which support robust and long-term efficacy in rodent and nonhuman primate brains upon direct cerebrospinal fluid (CSF) administration. Oligonucleotide distribution in the CNS is nonuniform, limiting clinical applications. The contribution of CSF infusion placement and dosing regimen on relative accumulation, specifically in the context of large animals, is not well characterized. To our knowledge, we report the first systemic, comparative study investigating the effects of 3 routes of administration — intrastriatal (i.s.), i.c.v., and intrathecal catheter to the cisterna magna (ITC) — and 2 dosing regimens — single and repetitive via an implanted reservoir device — on di-siRNA distribution and accumulation in the CNS of Dorset sheep. CSF injections (i.c.v. and ITC) resulted in similar distribution and accumulation across brain regions. Repeated dosing increased homogeneity, with greater relative deep brain accumulation. Conversely, i.s. administration supported region-specific delivery. These results suggest that dosing regimen, not CSF infusion placement, may equalize siRNA accumulation and efficacy throughout the brain. These findings inform the planning and execution of preclinical and clinical studies using siRNA therapeutics in the CNS.

## Graphical abstract

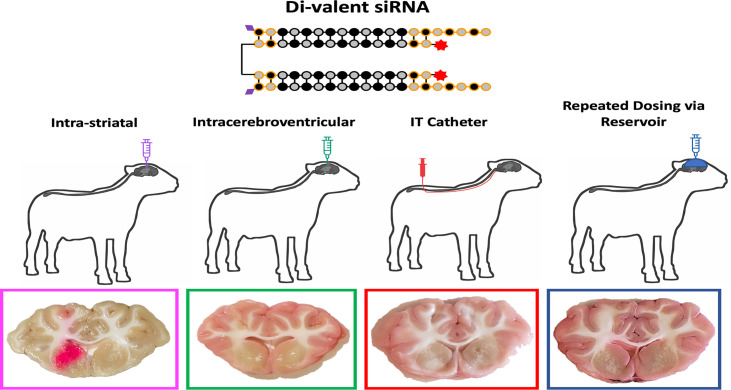

## Introduction

siRNAs and antisense oligonucleotide (ASO) drugs can be chemically engineered to silence almost any disease-linked gene and hold great potential for the treatment of neurological disorders. Therapeutic oligonucleotides cannot cross the blood brain barrier (1, 2) and, thus, require direct administration into the CNS to achieve potent and sustained gene silencing. Depending on the therapeutic strategy, siRNAs and ASOs can be administered directly into brain tissue via intraparenchymal injection or directly into cerebrospinal fluid (CSF) (1, 3, 4). There has been extensive investigation on the clearance and distribution of ASOs that are currently in clinical development but minimal evaluation of other oligonucleotide classes.

Evaluation of the pharmacokinetic (PK) and pharmacodynamic (PD) properties of ASOs targeting various genes in rats (GluR-1, Gabra1, Malat1) and nonhuman primates (NHPs) (Malat1) showed that intrathecally delivered (i.t.-delivered) ASOs distribute primarily to the cortex, with lower levels in deeper brain regions in both species (5, 6). At approximately 1 week after injection the maximum ASO accumulation was achieved in the cortex (~10 μg/g), which accounts for less than approximately 1.4% retention of the injected dose (0.7 mg) in the CNS. This supported robust silencing in the cortex, hippocampus, and spinal cord for at least 4 weeks (5), with minimal activity observed in the striatum. ASO accumulation and efficacy correlated well. Thus, early CSF-driven clearance kinetics may drive initial region-specific tissue accumulation that determines long-term efficacy.

CSF travels and distributes solutes throughout the brain via bulk flow (a) through the ventricular spaces, (b) down the spinal cord, and (c) through the glymphatic system (7, 8). Bulk CSF flow is likely the primary mechanism by which oligonucleotides distribute throughout the brain, and the ratio between the rate of oligonucleotide clearance from the CSF and CSF flow rate likely determines oligonucleotide distribution within the CNS (4). Mice and rats have a significantly smaller CSF volume and different flow rate kinetics than humans and NHPs (8). In larger species, it is thought that smaller solutes in CSF are cleared into the venous system through arachnoid granules, while larger macromolecules drain through the CNS lymphatic system, resulting in a wider variability in clearance time from the CSF and brain interstitium (9, 10).

Divalent siRNAs (di-siRNAs), which differ greatly in size from ASOs, result in robust CNS silencing throughout the NHP brain with variations in region-specific distribution (11). Di-siRNAs comprise 2 linked siRNA duplexes, generating a complex, approximately 27 kDa molecule, compared with approximately 7 kDa single-stranded ASOs. As both the size and the charge distribution of these 2 classes of oligonucleotides are different, better understanding of di-siRNA distribution between species is necessary.

The CNS distribution of di-siRNAs after i.c.v. injection varied between mice and NHPs (11). In mice, we observed broad distribution of di-siRNA throughout the entire brain. In NHPs, distribution was not uniform, with up to 4 times more di-siRNA accumulation in the cortex, hippocampus, and spinal cord compared with deeper brain regions, such as the caudate and putamen (11). Thus, the pattern of distribution of CSF-administered di-siRNAs in mice and rats may be different that in larger brains.

Oligonucleotides can be clinically administered into CSF via the ventricular spaces — i.e., i.c.v. injection (using an Ommaya reservoir) — or into the subarachnoid space of the spinal cord — i.e., direct i.t. injection (7, 12, 13). In addition to location, the rate of infusion can also be changed; oligonucleotides can be administered via a rapid bolus injection or a sustained administration over time, using a pump with bolus supporting better distribution (14). The size of the brain, the CSF volume, the CSF flow kinetics, and the oligonucleotide size may have varying effects on oligonucleotide distribution throughout the brain, depending on the route of administration (ROA) and dosing regimen. However, no side-by-side comparison between infusion placement, particularly for siRNAs, has been performed in multiple species. As the field of oligonucleotide therapeutics continues to grow and novel RNA-based therapeutics are approved for clinical use, distribution studies in larger brains are increasingly necessary.

Dorset sheep are a good model for investigating the impact of administration route on oligonucleotide distribution in CNS because of the large brain size (~140 g), spinal cord length (~50 cm), and CSF volume and flow that more readily reflect those of humans (15). Here, we compared the 3 most used administration methods in Dorset sheep — intrastriatal (i.s.; 1.6 mg), i.c.v. (50 mg), and i.t. catheter to the cisterna magna (ITC; 50 mg) — as well as 2 dosing regimens — single (i.c.v.) or weekly repeated dosing into the lateral ventricle through an implanted reservoir device (RD; 4 × 25 mg) — and evaluated the impact on distribution and efficacy 48 hours after injection. Intraparenchymal administration into the striatum supported local, region-specific delivery. The placement of CSF infusion (i.c.v. and ITC) had limited effect on siRNA distribution profiles; both showed large differences in accumulation between deep and cortical brain regions. Interestingly, repeated dosing increased accumulation in deeper brain regions, including caudate, putamen, and ventral hippocampus, without significantly affecting cortex accumulation, thus supporting more uniform distribution. These data confirm the substantial effect of brain size and CSF volume on siRNA distribution and highlight the relative importance of delivery route and dosing regimen on siRNA PK/PD within the CNS.

## Results

### Placement of CSF di-siRNA administration (i.c.v vs. i.t) substantially affects brain and spinal cord distribution in mice.

**Figure 1:**
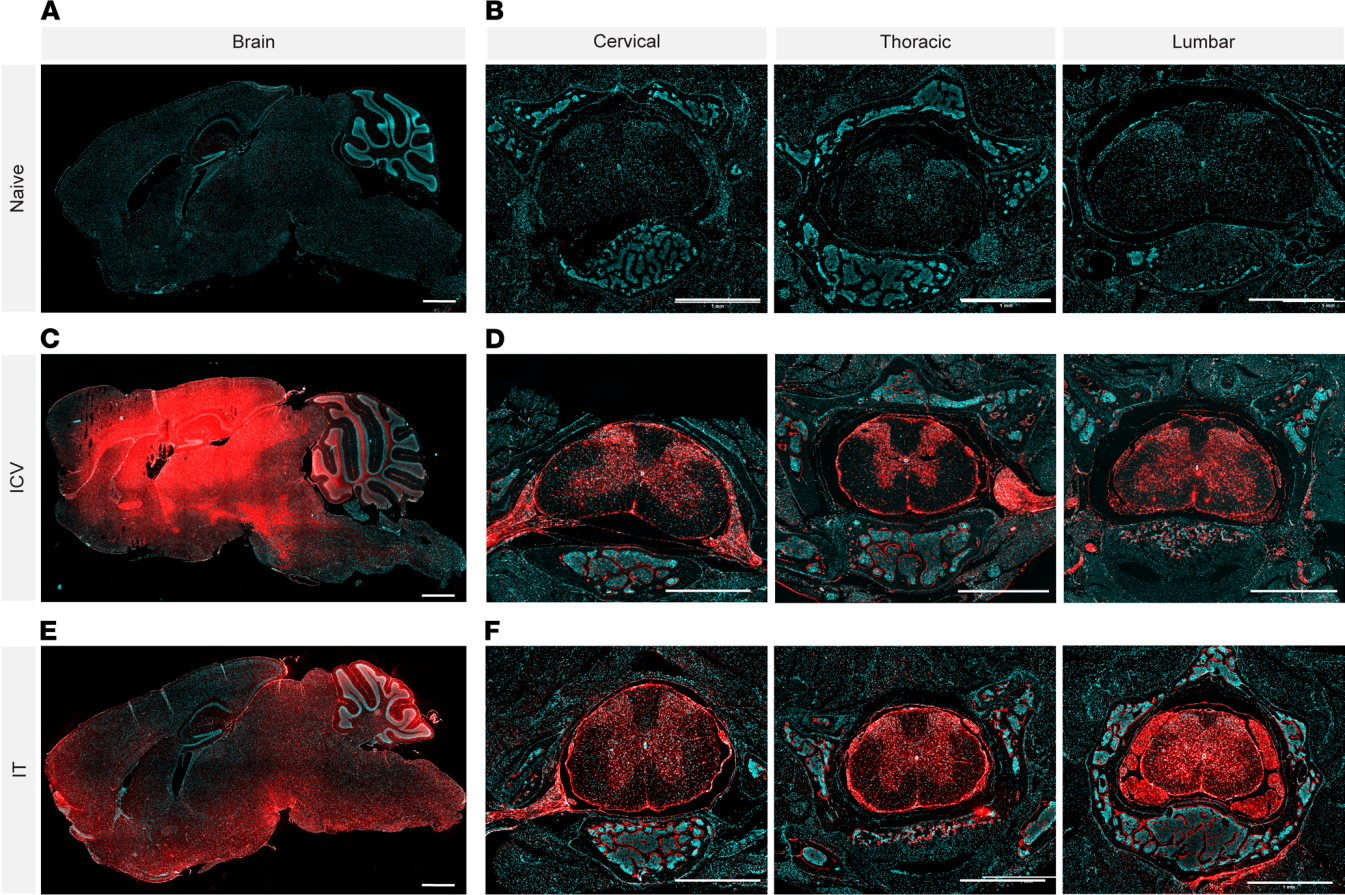
Placement of CSF di-siRNA administration (i. c.v. vs. i.t.) substantially affects brain and spinal cord distribution in mice. (**A**) Naive control showing DAPI staining (blue) in the brain. (**B**) Naive control showing DAPI staining (blue) in the cervical (right), thoracic (middle), and lumbar (left) spinal cord. Distribution of di-siRNA (red, Cy3) throughout the brain (**C**) and spinal cord (**D**) after direct i.c.v. administration. Distribution of di-siRNA (red, Cy3) throughout the brain (**E**) and spinal cord (**F**) after intrathecal administration. Original magnification, ×5. Scale bar 1 mm. *n* = 2 per group. Forty-eight-hour time point.

We previously demonstrated that i.c.v. administration of di-siRNA in mice resulted in broad distribution throughout the entire brain and spinal cord, including deeper brain regions such as striatum and hippocampus (11). However, i.t. injection may be the more clinically relevant and translatable approach. The impact of CSF infusion placement on di-siRNAs, which are much larger in size compared with ASOs, has not been reported. To investigate the relative impact of the CSF infusion placement (i.t. vs. i.c.v.) on di-siRNA distribution in the CNS in mice, 237 μg di-siRNA (Cy3, red) was injected into the lateral ventricles (i.c.v.) or into the i.t. space, and PBS was used as a control (Figure 1). Opposite distribution profiles were found when directly compared throughout the brain (Figure 1, A, C, and E). While i.c.v. administration resulted in more substantial distribution to the deeper brain structures, such as the caudate and putamen, i.t. administration showed preferential distribution to the cortex (Figure 1, C and E). Administration of both i.c.v. and i.t. enabled di-siRNA accumulation in the cerebellum and brain stem; however, compared with the deeper brain structures, the relative amount was greater in the cerebellum after i.t. administration (Figure 1, C and E). Distribution throughout all regions of the spinal cord (cervical, thoracic, and lumbar) was comparable between i.c.v. and i.t. administration (Figure 1, B, D, and F). Thus, in rodents, the placement of CSF infusion markedly affects distribution profiles. In models of disease in which cortex and spinal cord involvement predominates (i.e., ALS), both i.c.v. and i.t. administration are sufficient to target CNS regions of interest (16). For other diseases, such as Huntington disease where delivery to deep brain structures is essential, i.c.v. administration is preferred (5). Considering the technical challenges with i.t. administration in rodents, and widespread distribution observed with i.c.v., we have successfully used i.c.v. for di-siRNA administration in mice to target multiple genes (11). The clear differences in distribution between infusion placement in mice led us to investigate if CSF infusion placement would have similar effects in larger brains.

### Placement of CSF di-siRNA infusion (i.c.v. vs. ITC) does not significantly affect brain and spinal cord distribution in sheep.

**Figure 2:**
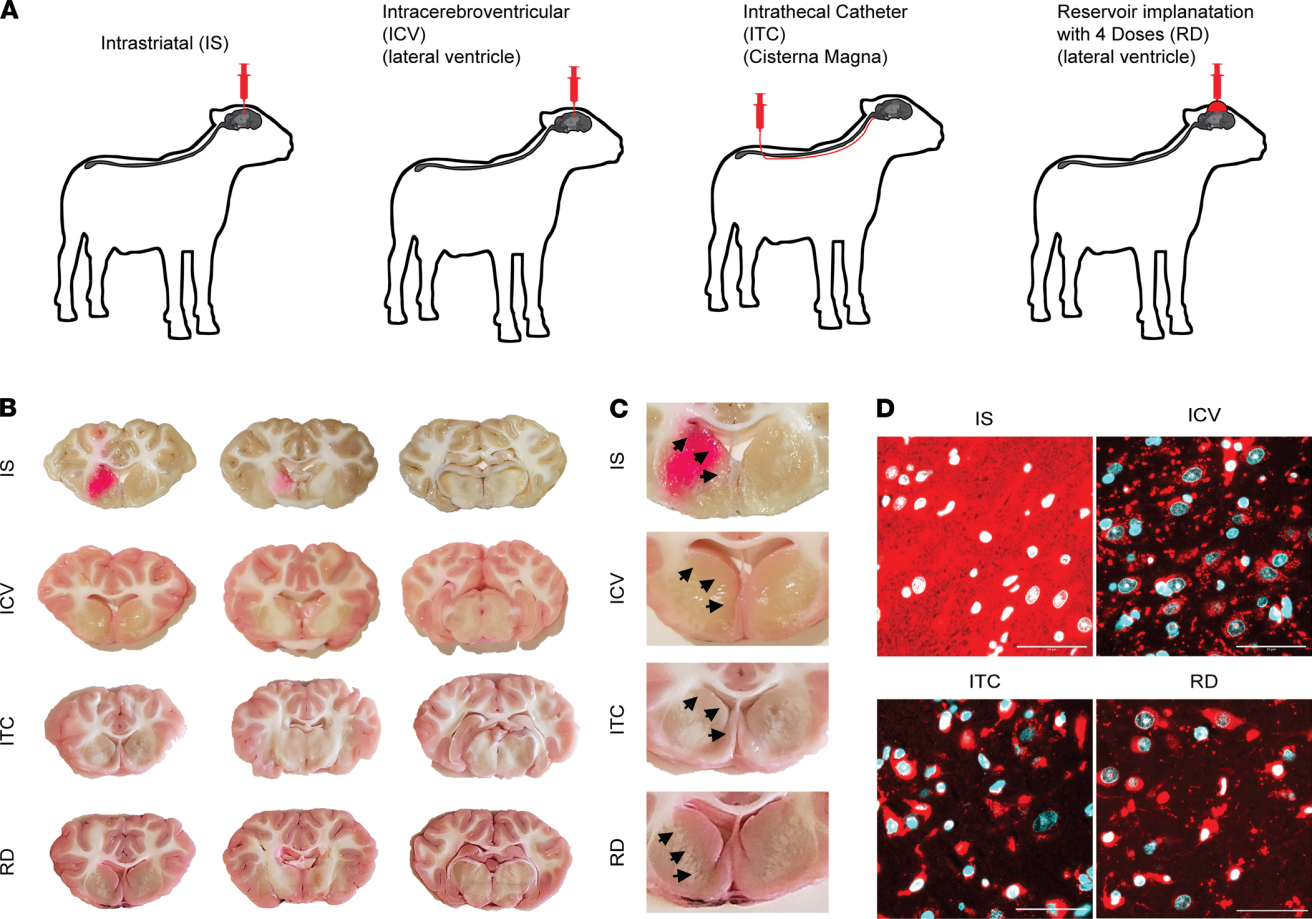
Administration of di-siRNA into the CSF results in widespread distribution throughout the CNS, including deep brain regions, in sheep. (**A**) Study design. (**B**) Gross sheep brain transverse images showing di-siRNA (pink) distribution after i.s., i.c.v., ITC, and reservoir route of administration. (**C**) Di-siRNA (pink) distribution to deeper brain regions, including the caudate (arrows) and putamen. (**D**) High-resolution images of caudate showing distribution of di-siRNA (red) to all cells in the caudate. Original magnification, ×63. Scale bar: 50 μm. *n* = 2 per group. Forty-eight-hour time point.

To determine the effect of CSF infusion placement on di-siRNA distribution in sheep CNSs, we administered di-siRNA to Dorset sheep via i.s. injection (1.6 mg), i.c.v. injection (unilateral, 50 mg), or injection via ITC (50 mg) (Figure 2A). The use of catheter limits the loss of material due to backflow often observed with direct lumber puncture injection in sheep and provides delivery closer to the brain. Forty-eight hours after treatment, we visualized the biodistribution of di-siRNA in the CNS using gross anatomical images and fluorescence microscopy.

After parenchymal administration into the striatum, gross anatomical images show local distribution of di-siRNA in the caudate and putamen, with limited spread of di-siRNA to other regions of the CNS (Figure 2, B–D). A single i.s. administration of approximately 50 μl di-siRNA resulted in distinct, local delivery, with approximately 6 mm diameter spread (Figure 2C), which, in sheep, covers the whole striatum. Thus, intraparenchymal administration of di-siRNAs enables region-specific delivery with limited accumulation in other brain regions. Although multiple local injections might be necessary to cover regions larger than approximately 6 mm^3^, the ability to drive siRNA delivery to specific brain regions holds many advantages in localized disease processes.

I.c.v. and i.t. injections in mice showed different distribution profiles in the brain (Figure 1). In sheep, both CSF infusion placements (i.c.v. and ITC) resulted in broad distribution throughout the brain (Figure 2B). While di-siRNA was detectable in deep brain structures, such as the caudate and putamen, the relative accumulation was substantially lower than in cortex (Figure 2, B and C). This observation is consistent with previously reported distribution of di-siRNA in NHP brains (11).

### Repetitive administration through Ommaya-like reservoir increases deep brain, but not cortex, accumulation of di-siRNA.

The loading dose is often harnessed to achieve steady-state drug concentration, and it is widely used for ASO (7, 17) and siRNA approved therapeutics (18, 19). Thus, we decided to evaluate if repetitive administration of di-siRNAs would be (a) well tolerated and (b) enhance deep brain accumulation. To enable minimally invasive repetitive administration in the sheep, we decided to use a sheep-compatible variant of the Ommaya reservoir. Ommaya reservoir devices are implanted under the skin or scalp and used to deliver therapeutic compounds directly into CSF (20–22). Ommaya reservoirs require a single surgery for implantation, after which the device can be used for repeat administration of therapeutics or removal of CSF without the need for anesthesia or a surgical procedure, allowing care takers to administer therapeutics in the outpatient setting (21). Ommaya reservoirs are mainly used for the administration of chemotherapy (22) but may be adapted for the repetitive administration of oligonucleotide therapeutics for the treatment of pain or neurodegenerative conditions.

Using a combination of MRI imaging, Brainsight technology (https://www.rogue-research.com/), and computerized tomography (CT) imaging, we surgically implanted an Ommaya-like device (reservoir connected to a catheter) into the right lateral ventricle of sheep. Correct placement of the catheter and reservoir was confirmed using CT images, in which the catheter was seen in the lateral ventricle, backflow of CSF occurred, and flow into the ventricular space after a gadolinium injection was visualized (Supplemental Figure 1; supplemental material available online with this article; https://doi.org/10.1172/jci.insight.152203DS1) (see Methods). After the placement of the reservoir, we were able to inject di-siRNA into the lateral ventricle with light sedation (Midazolam) at weekly intervals (25 mg once weekly for 4 weeks).

Repeated dosing through the reservoir was well tolerated with no observable adverse events or changes in blood chemistry profile (Supplemental Tables 1–3). The repetitive injection resulted in a visible increase in distribution to the caudate compared with i.c.v. or ITC injection (Figure 2, B–D). Although some delivery to the caudate was observed after single administration, the repetitive administration enhanced not only accumulation, but the depth of distribution away from the ventricles (Figure 2C) with delivery to all cell types observed (Figure 2D). Thus, loading dose may provide a path toward achieving enhanced di-siRNA delivery to deep brain regions.

**Figure 3:**
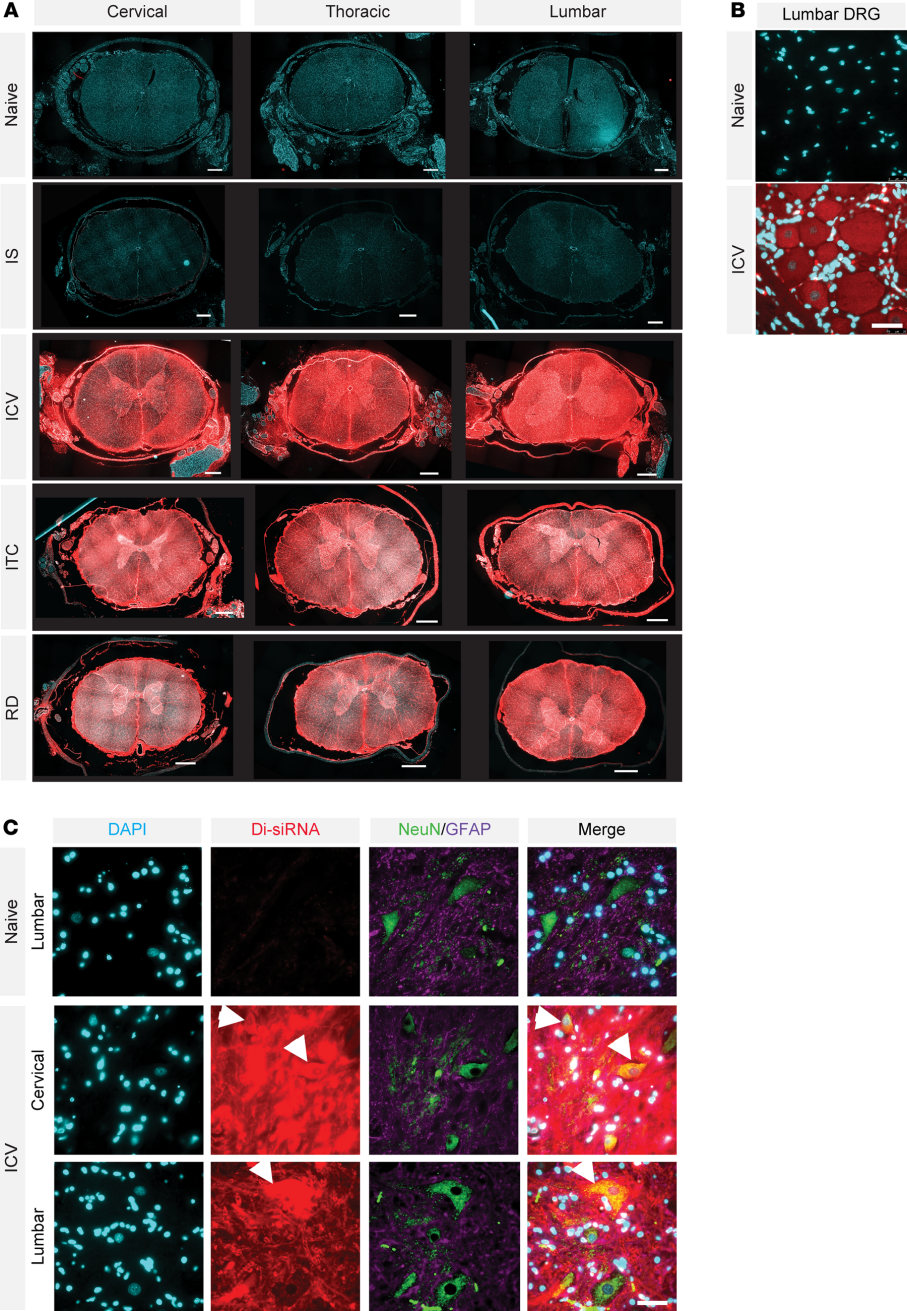
All ROAs into the CSF distribute throughout all regions of the spinal cord. (**A**) Visualization of di-siRNA (red, Cy3) distribution in the spinal cord. Original magnification, ×5. Scale bar: 1 mm. (**B**) Di-siRNA distribution to dorsal root ganglia in the lumbar region after i.c.v. injection. Scale bar 50 μm. (**C**) High-resolution images showing di-siRNA distribution to spinal motor neurons (green). Scale bar 50 μm.

No observable delivery to the spinal cord was seen after i.s. administration (Figure 3A). After CSF infusion (i.c.v., ITC, and RD), Cy-3–labeled di-siRNA was visible using fluorescence microscopy in all regions and levels of the spinal cord (Figure 3A). Fluorescence microscopy also showed that following i.c.v. administration, di-siRNA was taken up by dorsal root ganglia in the lumbar region (Figure 3B), motor neurons, and glial cells of the spinal cord (Figure 3C), suggesting that i.c.v.-injected siRNAs do not preferentially deliver to specific cell types and could be effective in treating neuromotor disorders like ALS.

### Di-siRNA distributes uniformly throughout the cortex after i.c.v. injection and is taken up by all cell types.

**Figure 4:**
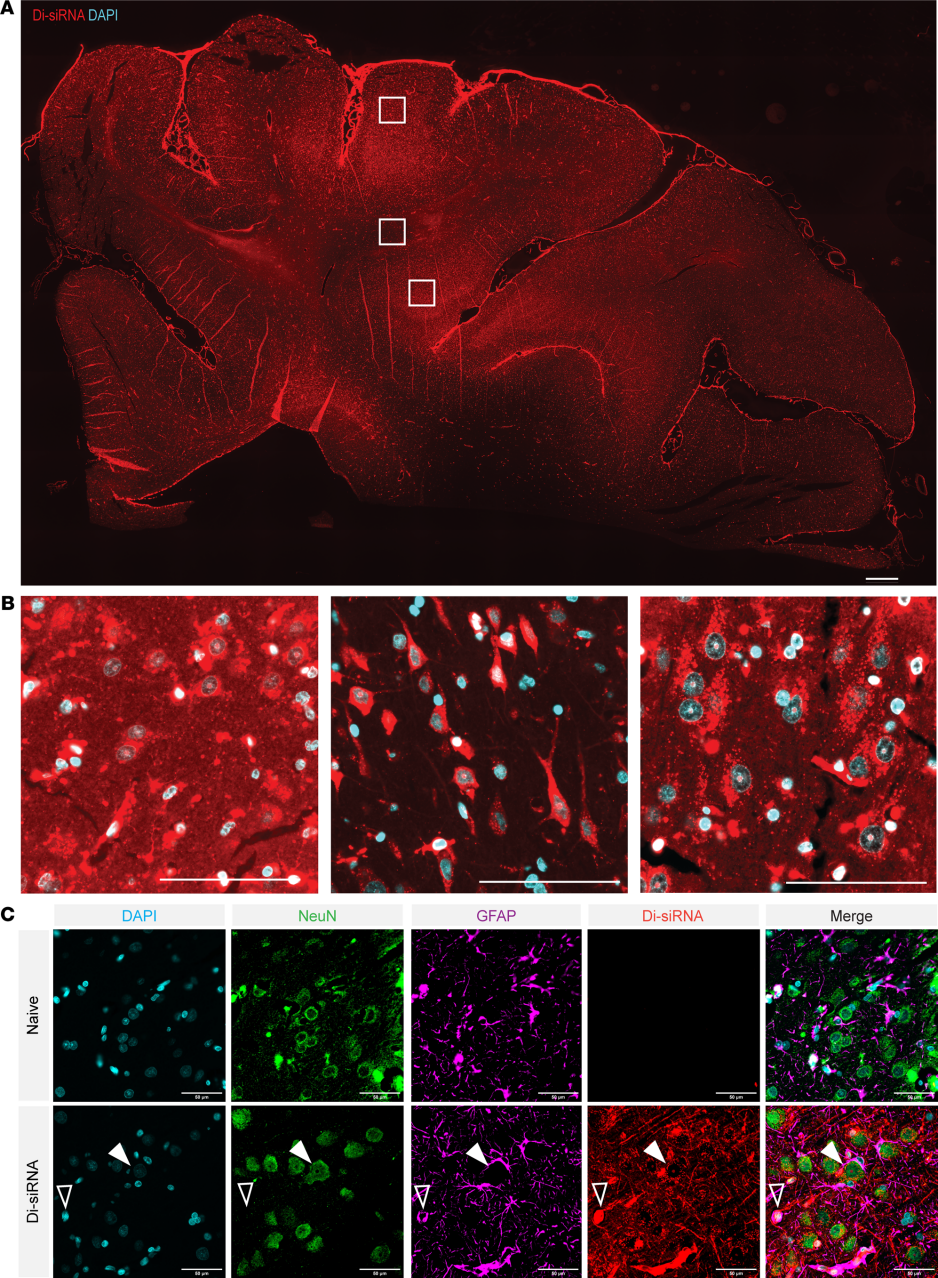
Uniform distribution of di-siRNA through all layers of the cortex after direct i. c.v. administration. (**A**) Distribution of di-siRNA (red) throughout the cortex of the sheep brain after direct i.c.v. administration. Original magnification, ×10. Scale bar: 1 mm. (**B**) High-resolution images of boxed regions (top, middle, and bottom boxes are displayed from left to right) showing even distribution of di-siRNA (red) to cell bodies in all regions, with less distribution to the pericellular areas in the deep cortex (middle) that contains more white matter. Original magnification, ×63. Scale bar: 100 μm. (**C**) Costaining of di-siRNA (red, Cy3) with neuronal (green, NeuN) and astrocytic (purple, GFAP) markers, showing delivery to both cell types in the cortex after i.c.v. administration. Original magnification, ×40. Scale bar: 50 μm.

While an accumulation differential was observed between deep brain structures and the cortex, di-siRNA distribution was uniform throughout all layers of the cortex, showing no indication of an accumulation gradient from superficial to deeper cortical layers (i.c.v. shown) (Figure 4, A and B), such as previously reported for ASOs (5, 23). Similar to ASOs, uptake of di-siRNA was observed with all cell types, including neuronal, glial, and epithelial cells (Figure 4, B and C). We observed diffuse uptake into glial cell bodies, glial cell projections (GFAP), and both perinuclear and cytoplasmic uptake in neurons (NeuN) (Figure 4C).

In summary, intraparenchymal administration of di-siRNAs limits distribution to an approximate 6 mm^3^ radius around the site of injection, with negligible overall CNS distribution. In contrast, both ITC and i.c.v. administration show similar spread throughout the CNS, with robust delivery to both the cortex and spinal cord, and less uptake in deep brain structures like the caudate and putamen.

### Repetitive CSF administration quantitatively enhances di-siRNA accumulation in deep brain structures without an increase in cortex accumulation.

While fluorescent imaging is useful in defining gross spatial distribution and confirming cell-type specific delivery, it is not sensitive enough to accurately quantify siRNA accumulation in tissue. To determine the effect of CSF infusion placement and dosing regimen on siRNA accumulation in the CNS, we quantified siRNA guide strand accumulation in each region of the brain and spinal cord at 48 hours after injection (final injection for RD group) using a previously validated peptide nucleic acid hybridization assay (24, 25). This assay detects the guide strand portion of the siRNA and is not dependent on the presence of the fluorescent label.

**Figure 5:**
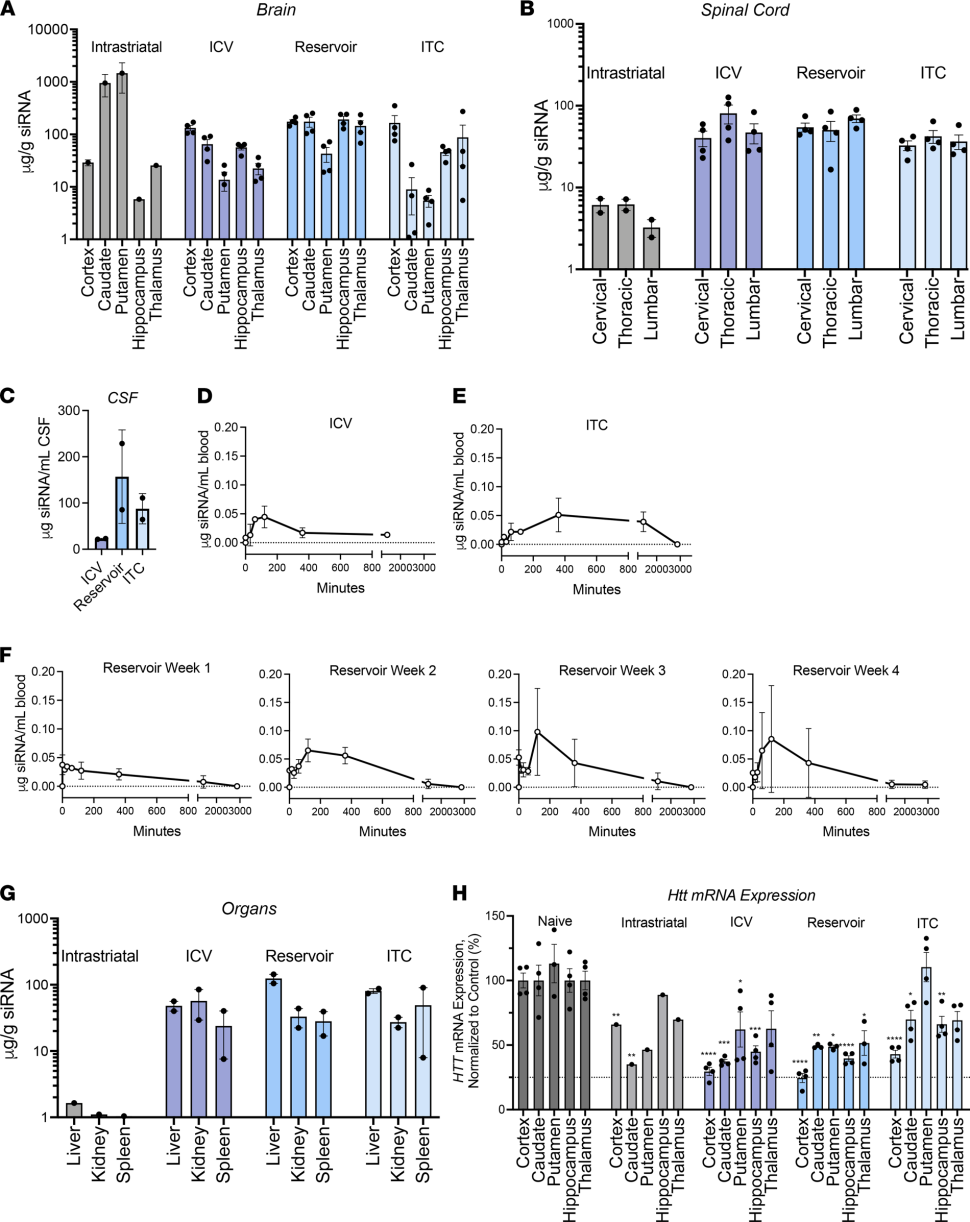
Guide strand accumulation, not silencing, increases with repeated dosing. Guide strand accumulation (μg siRNA/g tissue) throughout the (**A**) brain, (**B**) spinal cord, and (**C**) CSF (μg siRNA/mL CSF) 48 hours after final injection. (**D** and **E**) Guide strand accumulation in blood (μg siRNA/mL blood) for i.c.v. (**D**) and ITC (**E**) administrations. (**F**) Guide strand accumulation in blood (ng siRNA/μl blood) throughout the course of each RD administration (0–48 hours after injection). (**G**) Guide strand accumulation in clearance organs (liver, kidney, and spleen) 48 hours after injection. (**H**) mRNA silencing 48 hours after final injection throughout the brain after each ROA. Guide strand was quantified using PNA assay; mRNA was quantified using QuantiGene. *n* = 2 per ROA. *P < 0.1, **P < 0.01, ***P < 0.001, ****P < 0.0001, 1-way ANOVA. Data are shown as the mean ± SEM. Additional statistics are available in Supplemental Tables 4–9.

Overall, the results of quantitative guide strand accumulation studies were consistent with findings from gross anatomical images and fluorescence microscopy, showing increased distribution to deep brain areas with both single and repetitive i.c.v administration. After i.s. administration, we observed high siRNA accumulation in the caudate (~950 μg/g) and putamen (~1460 μg/g) regions immediately surrounding the point of injection (Figure 5A). Distribution to other parts of the brain was minimal, with substantially less accumulation in the cortex (~29 μg/g), hippocampus (~5.8 μg/g), and thalamus (~25 μg/g) (Figure 5A) and neglectable accumulation levels in the spinal cord (<5 μg/g) (Figure 5B). Quantification was done using standard curves specific for each region (see Methods) and levels below 1–3 μg/g approaches the background.

Consistent with fluorescent imaging, the i.c.v. and ITC administration supported high and uniform cortex accumulation (~130 μg/g), with lower accumulation in caudate (~65 μg/g for i.c.v. and ~9 μg/g for ITC) and putamen (~14 μg/g for i.c.v. and ~6 μg/g for ITC). Both i.c.v. and ITC administration resulted in accumulation in the hippocampus (~56 μg/g for i.c.v. and ~46 μg/g for ITC) and thalamus (~22 μg/g for i.c.v. and ~88 μg/g for ITC) (Figure 5A). While gross distribution was similar between CSF infusion placements, lower levels of accumulation were seen in deeper brain structures after ITC administration (Supplemental Tables 4 and 5).

The most interesting and unexpected results were observed with repeated dosing. Surprisingly, with repetitive dosing we did not observe significant differences in cortical (132 μg/g for i.c.v., 175 μg/g for RD) or spinal cord accumulation (Figure 5, A and B), even though the cumulative dose was 2-fold higher. In contrast, compared with a single i.c.v. injection, repeated dosing in the lateral ventricles led to an increase in di-siRNA distribution to the caudate (175 μg/g, 2.7-fold increase over i.c.v.; *P* = 0.0405), putamen (~20.5 μg/g, 1.5-fold increase; *P* = 0.1394), and hippocampus (192 μg/g, 3.4-fold increase; *P* = 0.0016) (Figure 5A) and overall, more uniform distribution throughout all brain regions (Supplemental Tables 4 and 5). The more uniform distribution observed with the repetitive administration is a very interesting trend and is worth further investigation.

### Changing CSF infusion placement has a limited impact on di-siRNA initial systemic clearance.

Our IACUC protocol limited repetitive CSF draws, thus the level of di-siRNAs circulating in CSF was measured at 48 hours after injection, the time of study termination. The level of di-siRNA in the CSF was relatively high 48 hours after injection (22 μg/ml for i.c.v.; ~87 μg/ml ITC; and 157 μg /ml RD [after last dose]) (Figure 5C), with 2%–16% of total injected dose still circulating in CSF (Supplemental Tables 6 and 7), indicating significantly slower CSF clearance compared with other classes of oligonucleotides.

Evaluation of di-siRNAs in the blood in the peri- and postoperative (48 hours after injection) periods showed similar trends between i.c.v. and ITC, although maximum levels of blood di-siRNAs were achieved at approximately 2 hours with i.c.v. and approximately 6 hours with ITC injections (Figure 5, D and E). The overall blood levels were relatively low (maximum less than <0.1 μg/ml), likely due to the combination of lower level of CSF clearance and quick uptake into clearance organs upon systemic exposure. The observed difference in early time points of i.c.v. and ITC kinetics needs further evaluation and might be indicative of variations in CSF clearance kinetics, or an artifact of surgical procedure, where higher levels of backflow might be occurring with i.c.v. versus ITC. The levels of circulating siRNA dropped to limit of assay detection by 48 hours (Figure 5, D and E, 48-hour data point missing for i.c.v.), indicating that by 48 hours, the majority of the siRNA has distributed to either the CNS or the clearance organs.

The overall clearance profiles were similar after the first, second, third, and fourth repetitive injection, with circulating siRNA levels falling below the limit of detection by 48 hours (Figure 5F). The maximum blood concentration was achieved sooner and was slightly lower for the first administration compared with the other 3 injections (Figure 5F). Once again, these differences could be related to procedural variations during administration. The first reservoir administration was performed during the surgical implantation, while the other 3 were done using the established device in slightly anesthetized animals.

As expected from PK/PD results in rodents (11), CSF administration of di-siRNAs resulted in accumulation in clearance organs, including the liver, kidney, and spleen (Figure 5G, Supplemental Figure 2, and Supplemental Table 8). After i.s. administration, we observed minimal accumulation in the clearance organs, likely due to the lower dose administered (1.6 mg vs. 50 mg) and the high retention of siRNA at the site of injection. Overall, accumulation in clearance tissues was not significantly affected by CSF injection placement, with the highest accumulation in the liver, followed by the cortex of the kidneys, and the spleen. The cellular pattern of distribution was similar to that of other oligonucleotides: siRNA accumulated uniformly in the liver, with some preferential delivery to Kupfer cells, and preferential accumulation in the proximal epithelia with limited uptake in the glomerulus and medulla, similar to the clearance distribution previously observed in rodents (Supplemental Figure 2) (11).

Thus, CSF infusion placement (i.c.v. vs. ITC) has a limited impact on overall distribution profile. In contrast, repetitive administration increased relative accumulation of di-siRNA in deep brain structures, without increasing cortex and spinal cord levels. Taken together, repeated dosing seems to result in more uniform distribution throughout all brain regions, potentially by saturating more accessible cortical regions. This phenomenon is not unique to the brain and has been previously shown to enhance the extrahepatic delivery of lipid-conjugated siRNAs after systemic administration (18).

### Administration of di-siRNA into the CSF supports HTT mRNA silencing as early as 48 hours of administration.

The targeting sequence used in these experiments has homology among mice, sheep, NHPs, and humans, and it targets huntingtin, the gene involved in Huntington disease (26, 27). While the primary goal of this study was to evaluate the feasibility and safety of different routes of administration (ROAs) on distribution, sequence homology allowed us to also evaluate the impact of ROAs on mRNA silencing throughout the brain, using naive animals as controls. It is important to note that with the small sample size and a less than ideal 48-hour time point (silencing may take weeks to reach its maximum), these results should be considered with care.

We evaluated silencing ipsilateral and contralateral to the site of injection and observed similar overall results between sides. Statistical analysis was performed using 1-way ANOVA and comparing the values from each brain region to those of the corresponding naive controls. Direct i.s. injection resulted in approximately 70% silencing in the ipsilateral caudate and putamen (*P* < 0.01 for putamen) and a trend toward approximately 30% reduction in the ipsilateral cortex (*P* < 0.01) and thalamus (not significant), and no silencing on the contralateral side (Figure 5H).

CSF infusion supported statistically significant silencing in most brain regions with all ROAs (Figure 5C), including cortex (i.c.v., 64%; ITC, 59%; RD, 72%; *P* < 0.01), hippocampus (i.c.v., 54%; ITC, 45%; RD, 60%; *P* < 0.01), and caudate (i.c.v., 70%; ITC, 54%; RD, 55%; *P* < 0.01). In the putamen, we only observed significant mRNA silencing with i.c.v. and repeated administration (45% and 50%, respectively), but ITC had no detectable activity (Figure 5C and Supplemental Table 9).

**Figure 6:**
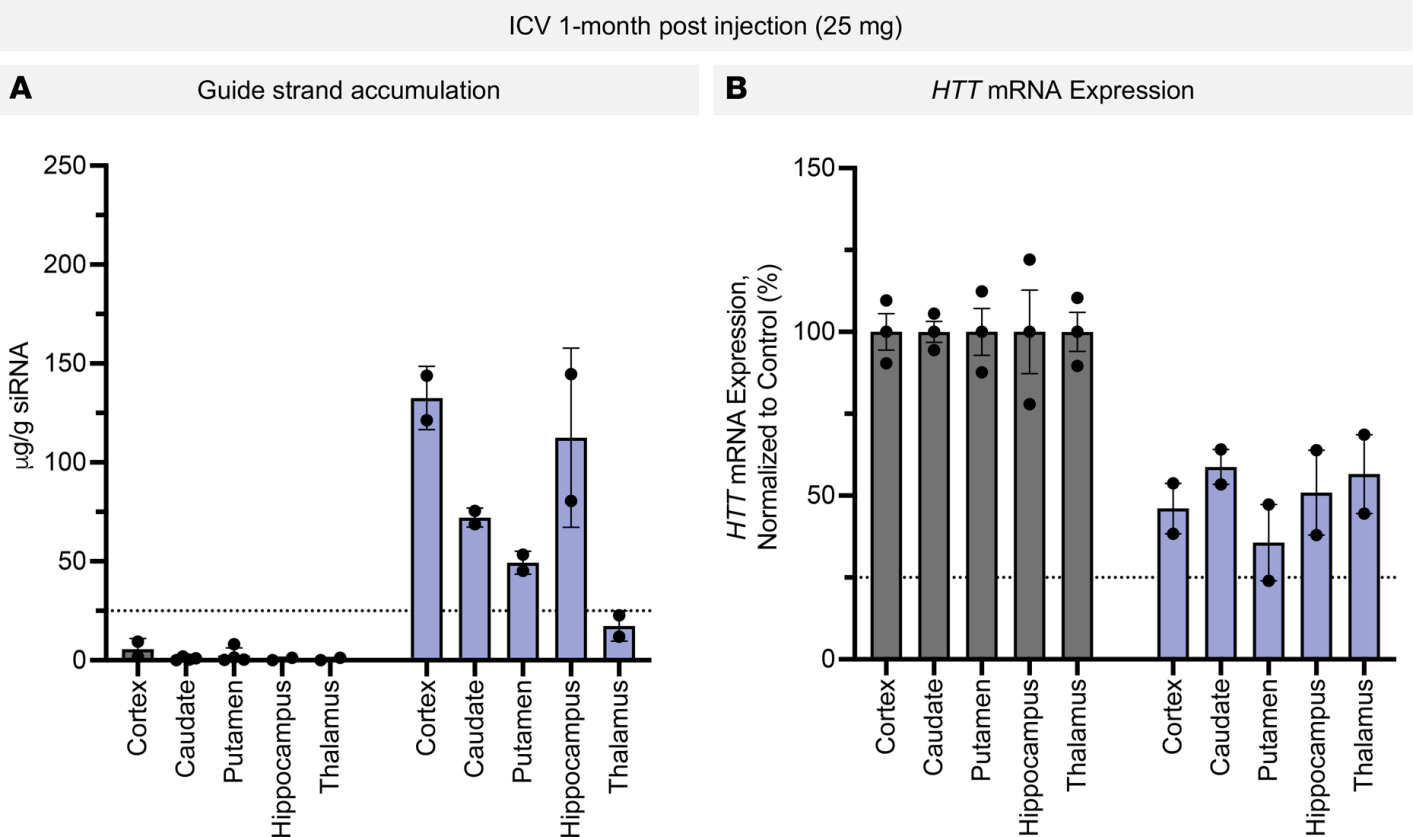
Sustained guide strand accumulation and mRNA silencing 1 month after i. c.v. administration. (**A**) Guide strand accumulation (μg siRNA/g tissue) throughout the brain 1 month after i.c.v. administration of di-siRNA (25 mg). (**B**) Target mRNA silencing 1 month after injection throughout the brain after each i.c.v. administration of di-siRNA. ROA was i.c.v.; a dose of 25 mg was used. One-month time point. *n* = 2. Guide strand was quantified using PNA assay; mRNA was quantified using QuantiGene. *n* = 2 per ROA.

To understand the longer-term distribution and efficacy, we evaluated guide strand accumulation and mRNA silencing in animals treated with 1 dose of 25 mg di-siRNA (i.c.v.) for 1 month. In the cortex, we observed an average of 132.6 μg/g siRNA, 72.14 μg/g in the caudate, 56.44 μg/g in the putamen, and 112.5 μg/g in the hippocampus (Figure 6A). At 1 month after injection, we observed sustained mRNA silencing in the cortex, caudate (42%), putamen (75%), hippocampus (50%), and thalamus (44%), even at a lower dose (25 vs. 50 mg), suggesting sustained duration of effect after a single dose (Figure 6B).

All ROAs tested had no appreciable systemic toxic effects and no changes from baseline in a panel of blood chemistries (Supplemental Tables 1 and 2). For RD, we also observed no significant changes in complete blood counts (Supplemental Table 3).

## Discussion

Here, we performed a systematic comparison of 4 routes of administration (i.s., i.c.v., ITC, and reservoir) of di-siRNA, a CNS-active siRNA scaffold, and evaluated the impact on distribution and silencing efficacy 48 hours after injection. This is the first side-by-side comparative study investigating the impact of infusion location on di-siRNA distribution and accumulation in the CNS of a large animal.

The placement of CSF injection in Dorset sheep (i.c.v. vs. ITC) had a minimal impact on overall distribution profiles. We observed typical, nonuniform accumulation in superficial and deep brain structures. While level of accumulation was lower in deeper brain structures, it was sufficient to support productive silencing of a therapeutically relevant target (*HTT*) as early as 48 hours after injection.

The most interesting observation of the study is the improvement in the relative deep brain accumulation after repetitive dosing (loading dose) that was achieved without a simultaneous increase in cortex and spinal cord accumulation. It is possible that the early doses in the repetitive regimen allowed the brain to be exposed to siRNA for longer. Longer exposure may lead to an additive effect and continued diffusion of siRNA throughout deep brain regions. In this study, di-siRNAs were injected weekly, and further optimization of exact dosing regimen is necessary. Furthermore, this study shows a benefit of loading doses to the ventricles via a subcutaneous reservoir and demonstrates an ability to do multiple noninvasive administrations for long term studies, which may define a path toward a therapeutic approach that ensures more uniform brain uptake. Overall, this work begins to address one of the most clinically relevant questions in the development of oligonucleotides for CNS therapeutics: what is the optimal route and dosing regimen of administration?

Interestingly, the relative impact of i.c.v. vs. ITC seems to be species specific. In mice (brain size of 0.4 g vs. ~140 g in sheep), the relative difference is substantial, with i.c.v. resulting in greater distribution throughout the deeper brain regions and cortex, while i.t. administration resulted in less distribution to deeper brain regions. In sheep, the relative impact of i.c.v. versus ITC is low, suggesting that varying the location of CSF administration does not greatly affect distribution in this species. Thus, it is likely that variation in brain size and CSF flow kinetics between species results in differences in siRNA distribution in the CNS.

In mice, i.c.v. is the preferred ROA given its reliable and reproducible distribution throughout the CNS in comparison to i.t. administration. In rats, which possess a brain size (2 g) only slightly larger than mice, oligonucleotide distribution is less variable after i.t. administration than in mice (5). It is possible that as the brain size and CSF volume increases, the impact of CSF infusion placement on distribution is less severe. Thus, in the context of other large animals (sheep, NHPs) and humans, varying CSF infusion placement may have a less discernible impact on distribution profiles.

Here, we show that direct i.s. administration results in local distribution of approximately 0.6 cm^3^ and results in potent silencing in a region-selective fashion (28). Thus, direct intraparenchymal injection might be of interest as a therapeutic ROA for solid tumors. Additionally, this ROA might be useful to perform functional brain region studies where regional gene expression may determine phenotypes but is not valuable for treating progressive and diffuse neurodegenerative disorders.

Surprisingly, repeated dosing via an implanted reservoir did not result in increased accumulation in the cortex or spinal cord compared with i.c.v. or ITC administration, but it did result in increased accumulation in deeper brain regions and more consistent accumulation between brain regions. Similarly, repeated dosing did not significantly increase mRNA silencing in the cortex, putamen, or caudate, but did increase silencing in the hippocampus and thalamus. It is possible that, despite administering twice the dose, accumulation and silencing in these regions reached saturation with a single administration. We hypothesize that the increased spread into deeper brain regions occurs after cortical regions are saturated, in a mechanism like that observed systemically to achieve extrahepatic delivery of lipid conjugated (17). Therefore, loading doses may be more clinically relevant for diseases that affect deeper brain structures in addition to the cortical regions. The phenomenon of tissue and cellular saturation is widely observed in the field of oligonucleotides, but its etiology is not well understood (17). For example, repetitive systemic administration of docosahexaenoic acid conjugates siRNA shows variable relative increases in accumulation for different tissue (17). The heart is completely saturated with a single 20 mg/kg dose, and 5 additional injections within 3 days does not result in any additional uptake. In contrast, a second injection (within 12 hours) results in 2-fold increase in liver accumulation, while it took additional doses and a longer duration to saturate muscle accumulation (17). Thus, repetitive dosing, and saturation of tissue accumulation in certain regions, might provide a safe and efficient way to unify siRNA brain distribution, a currently unaddressed and critical challenge. The duration of effect of siRNAs is strongly correlated to the level of initial siRNA accumulation; thus unequal initial accumulation is likely to result in variable duration of effect, as observed between different regions. Consistent with this, after 1 month, there was less di-siRNA accumulation and, similarly, less mRNA silencing after a single i.c.v. injection of 25 mg. Future studies evaluating duration of effect for different doses and dosing regimens are necessary to truly understand the impact on silencing duration.

CSF facilitates exchange through multiple flow routes — it travels down the spinal cord, and it enters the glymphatic space and the subarachnoid space (29, 30). As CSF circulates through these routes, it continuously bathes the cortical surface of the brain; additionally, other CSF routes interact more directly with deeper brain regions (31). For all ROAs into CSF, di-siRNA is clearly visible in ependymal cells on the ventricular surface and the spinal canal, as well as in all layers of the cortex. Although it is possible that some delivery is due to diffusion-based uptake on the surface of the cortex and ventricles, observed delivery to all layers of the cortex after administration into CSF (i.c.v., ITC, RD) supports the hypothesis that di-siRNA is entering brain tissue and cells via multiple CSF circulatory routes, including the flow of CSF around the brain and spinal cord and through the glymphatic system. Moreover, the sustained presence of di-siRNA in the CSF suggests that it is not being cleared by the traditional routes that are thought to clear ASOs rapidly — e.g., via arachnoid villi into the venous system (9). After administration into the CSF space, we hypothesize that di-siRNA remains in the CSF due to its characteristically large size and charge (~27 kDa vs. ~7 kDa for ASOs) (11), which may slow down its rapid clearance into the venous system.

It is also possible that di-siRNAs are cleared more slowly through CNS glymphatics and lymphatics, such as other macromolecules, and that this phenomenon is enhanced with repeated dosing (9, 32, 33). It has been shown in rats that the mean solute speed of glymphatic flow is greater in the hippocampus and posterior cortex than in caudate and putamen (34). Glymphatic flow directly reflects the distribution we observe of di-siRNAs throughout the brain after direct injection into CSF. Thus, further understanding of how glymphatic flow contributes to oligonucleotide distribution is necessary in future studies.

Assuming the CSF volume is 25 ml in sheep, between 2% and 16% of the injected dose is still circulating in the CSF 48 hours after injection. The highest amount observed is after repetitive administration, indicating that the longer duration of the study might have supported increased deep brain accumulation. The high level of siRNA observed in the CSF 48 hours after injection was somewhat surprising. The PK/PD of ASOs in the CNS is well understood and characterized. Following CSF injection, ASO CSF levels drop exponentially, with residual levels dropping way below the fraction of injected dose within 24 hours (4, 14). We hypothesize that the large size is one of the primary drivers behind slower di-siRNA clearance kinetics, higher CSF retention, and higher levels of deep brain accumulation.

The differences in CSF retention between di-siRNAs and ASOs are also observed in early blood levels. The highest blood concentration for di-siRNA never exceeds approximately 0.1 μg/ml, which is lower than levels observed with ASO compounds in range of reported studies (4). While overall distribution and clearance rate are similar between ASO and siRNA, the siRNAs may display trends toward slower clearance.

Supplemental Table 7 shows estimates of the percentage tissue retention of di-siRNA in different brain regions, spinal cord, CSF, and clearance tissues (liver, kidneys, and spleen). Between CNS and clearance tissues, we can account for 75% to 100% of injected dose, indicating a high degree of bioavailability. We estimate that after 48 hours, between 33% and 43 % of injected dose is retained in CNS tissues, from which 20%–36% is accumulated in the cortex, 0.02%–0.14% in striatum, 0.9%–1.7% in the spinal cord. The sheep cortex makes up the largest fraction of sheep brain driving high percentage of retention. The high level of brain retention is likely to support sustained duration of silencing for many months (5, 6, 11). Clearance profiles are similar between different routes of administration, with liver and kidney accounting for majority of systemically distributed dose (32%–54% in liver) and (3%–12% in kidneys). Thus, liver and kidneys are two tissues which need to be evaluated carefully in long-term repetitive dosing safety studies.

An additional factor to consider for optimal infusion placement is the effect of disease on brain anatomy and structure. As neurodegeneration persists, the brain undergoes significant cortical and striatal (in the case of Huntington’s disease) atrophy. When brain parenchyma atrophies, the ventricular size increases, and the CSF flow dynamics change (29, 31, 35, 36). Thus, oligonucleotide distribution might change in diseased or aged states. In the context of diseases that affect the cortex and striatum, such as Huntington’s disease, a repetitive dosing paradigm which would increase relative caudate and putamen accumulation might be essential to achieve a clinically relevant therapeutic response. Alternatively, for conditions that initiate in the hippocampus and affect the hippocampus and cortex, such as Alzheimer disease, or diseases that mainly affect spinal cord and cortex, i.t. administration may be sufficient.

One limitation of our current study is that it evaluates distribution and efficacy at a single time point for all ROAs tested. It is likely that distribution of di-siRNA in large animals changes over time, as in rodents (5). While the degree of mRNA silencing has likely not reached its maximum or steady-state amount at 48 hours after injection, confirmation of silencing 1 month after injection (11) suggests a rapid onset of silencing efficacy followed by long-term sustained silencing. Although exciting, this study only scratches the surface — further evaluation of the mechanisms behind the impact of ROA and dosing regimen on distribution and efficacy at multiple time points is necessary.

Overall, this study shows both the feasibility and the impact of varying CNS infusion placement for oligonucleotides, specifically siRNA, and serves as a useful resource to guide future experiments and the clinical development of therapeutic siRNAs. We show that no single route of oligonucleotide administration is optimal for all clinical entities. Instead, understanding how each ROA affects distribution will be useful for selecting the most appropriate route and dosing regimen for each clinical indication.

## Methods

### Oligo production.

Oligonucleotides were manufactured as described in Alterman et al. (11). The following modified antisense sequence was used: V(mU)#(fU)#(mA) (fA) (mU) (fC) (mU) (fC) (mU) (fU) (mU) (fA) (mC)#(fU)#(mG)#(fA)#(mU)#(fA)#(mU)#(fA), where “#” denotes phosphorothioate linkage and “m” and “f” denote modification of the endogenous 2′OH group to 2′ O-methyl or 2′ fluoro, respectively. The following modified sense sequence was used: Cy3-(fC)#(mA)#(fG) (mU) (fA) (mA) (fA) (mG) (fA) (mG) (fA) (mU) (fU)#(mA)#(fA)-DIO.

### Mouse i.c.v. injections.

Mice were anesthetized using avertin (i.p. injection). For i.c.v. injections, 10 μl di-siRNA was administered bilaterally (5 μl per ventricle) into the lateral ventricles of mice as previously described (11). Stereotaxic devices were using to hold injection needles and identify injection location. After the identification of the bregma, the needle was placed 1 mm laterally, 0.2 mm posterior, and 2.5 mm caudally. Injection was performed at 500 nl/min. Mice were then monitored until fully sternal.

### Mouse i.t. injections.

Animals were anesthetized using isoflurane and placed on a nose cone with constant flow of isoflurane. Animals were shaved near the base of the tail to facilitate a better visualization during needle insertion. L5–L6 was visualized, and the needle was carefully inserted between the groove of L5 and L6 vertebrae. Tail flick indicated a successful entry of the needle into the intradural space. 10 μL of the test article was administered slowly. Animal was returned to its cage and monitored for pain and distress.

### I.t. catheter injections.

Animals were anesthetized using ketamine and diazepam, intubated, and maintained with isoflurane and propofol. Access to the i.t. space was obtained using standard lumbar puncture technique at L7/S1. A catheter was threaded through the i.t. space to the cisterna magna using fluoroscopy guidance (Azurion, Philips Medical Systems). Location was guided and confirmed by a combination of cone beam CT (Xper FD20 System), fluoroscopy, and x-ray imaging after iodinated contrast injection (Omnipaque 240 mg/mL GE Healthcare). 50 mg di-siRNA in 1.5 ml was injected at a rate of 1 mL/min.

### Sheep surgical procedures.

Sheep were anesthetized as previously described. The area over the frontal bone was clipped and aseptically prepared using standard techniques. An incision was made over the frontal bone and an MRI fiducial array was fixed to the skull. Animals then underwent 3T MRI (3T Phillips Ingenia) to generate 3-dimensional T1 weighted images for neuro-navigation (MPRAGE 0.5 mm isotropic voxels). The sheep were then placed in a radiolucent stereotaxic frame (Model 1530M, Khopf instruments) and registered to neuro-navigation equipment (Brainsight Vet, Rogue Research). The head of the sheep was shaved and aseptically prepped using standard techniques. A lateral incision was made, the skull exposed and craniotomy made based on neuro-navigation MRI coordinates.

### I.c.v. injections.

MRI-based neuro-navigation was used to determine the location and trajectory to access the lateral ventricles. Using neuro-navigation MRI guidance, a spinal needle (17 gauge) was lowered into the brain using the stereotaxic manipulator. Backflow of CSF was used to confirm the location in the right lateral ventricle. mg of di-siRNA in 1.5 ml was injected at a rate of 50 μl per minute.

### Reservoir implantation and repetitive dosing.

Sheep were anesthetized as previously described. Access to lateral ventricles was found and insertion of spinal needle was done as previously described. Once the needle was in place, a custom-made radio-opaque flexible catheter (5 French; SAI infusion Technologies) was lowered into the lateral ventricle, and CSF backflow was used to confirm location. The catheter was secured to the skull using a combination of bone cement and subcutaneous sutures. The catheter was then attached to the reservoir SAI infusion Technologies) with some slack to allow for small movements. Placement was verified by fluoroscopy and cone beam computed tomography after injection of iodinated contrast (Omnipaque 240 mg/mL). Both the catheter and the reservoir were placed under the skin, and the incision was closed using standard techniques. Once a week for 4 weeks sheep received 25 mg di-siRNA in 1.5 ml at a rate of 50 μl/min. Sheep were lightly anesthetized using midazolam and xylazine and then received isoflurane for the duration of the injection. The reservoir was identified under the skin, and the injection site was cleaned according to standard aseptic procedure. Patency of the catheter was confirmed, and injection was performed using a noncoring Huber needle. Animals were allowed to recover from anesthesia and returned to pen when fully awake and sternal.

### Necropsy.

All animals were euthanized 48 hours after final injection by pentobarbital overdose (Fatal Plus, 120 mg/kg). Animals were perfused via the carotid arteries for 1 hour with 20 L of PBS at a rate of 400 ml/min. Brain was removed from skull and sliced using Brain Matrix (a custom-made brain matrix, manufactured at University of Massachusetts Medical School) at 4 mm intervals. Tissues were collected and stored in either formalin or RNAlater for processing.

### Microscopy.

Distribution of di-siRNA was visualized using Cy3 dye conjugated to the siRNA molecule. Tissue was fixed in 10% neutral buffered formalin, paraffin embedded, and sliced into 10 μm slices. Slices were deparaffinized in 100% xylene for 8 minutes; rehydrated in 100%, 95%, and 80% ethanol for 4 minutes each; washed twice in 1X PBS for 1 minute; and placed in DAPI (1:10,000) for 2 minutes. Neurons were labeled with anti-NeuN antibody (1:300) (MAB377) and astrocytes were labeled with anti-GFAP antibody (1:1000) (Agilent GA52461-2).

### Quantification of guide strand accumulation.

2 mm punches were taken from each brain region and homogenized in homogenizing solution (Thermo Fisher Scientific) with proteinase K and incubated at 55°C. Three punches were taken from the cortex, 2 from the caudate and putamen, and 1 from the hippocampus and thalamus per animal per side of the brain. Guide strand accumulation was quantified in each sample using PNA hybridization assay as previously described in Godinho et al. (25). Each dot corresponds to the average value of any technical replicates.

### mRNA silencing.

mRNA was quantified using the QuantiGene assay (Thermo Fisher Scientific) and probes specific for sheep huntingtin (*HTT*) mRNA. Briefly, punches were homogenized in homogenizing buffer with proteinase K (1:100) and incubated at 65°C for 1 hour. Sheep HPRT and PPIB were used as housekeeping mRNA. Samples were normalized to naive controls.

### Statistics.

All statistics were performed and analyzed with GraphPad Prism. Target silencing was analyzed using 1-way ANOVAs with Tukey’s corrections, comparing the mean of every column to each other. *P* values of less than 0.05 were considered significant.

### Study approval.

All experimental studies involving animals were approved by the University of Massachusetts Medical School IACUC (protocol #A-2411 and #A-2593) and performed according to the guidelines and regulations therein described.

## Author contributions

AK, NA, JFA, BMDCG, and CMF conceived of the project. AK, NA, HGE, MJG, JFA, BMDCG, CMF, AHC, and RPM contributed to experimental design. CMF, BMDCG, JFA, AHC, EGK, HGE, MJG, RPM, RMK, TT, AP, JC, RAH, and JWG contributed experimentally. MH and DE synthesized compounds. CMF and AK wrote the manuscript.

## Supplementary Material

Supplemental data

## Version 1 12/22/2021

Electronic publication
