# Supplementary material for: Comparative route of administration studies using therapeutic siRNAs show widespread gene modulation in Dorset sheep
Source: JCI Insight. 2021 Dec 22;6(24):e152203. doi: 10.1172/jci.insight.152203 (PMC8783676; doi:10.1172/jci.insight.152203)
Supplement: Supplemental data [file jciinsight-6-152203-s237.pdf]

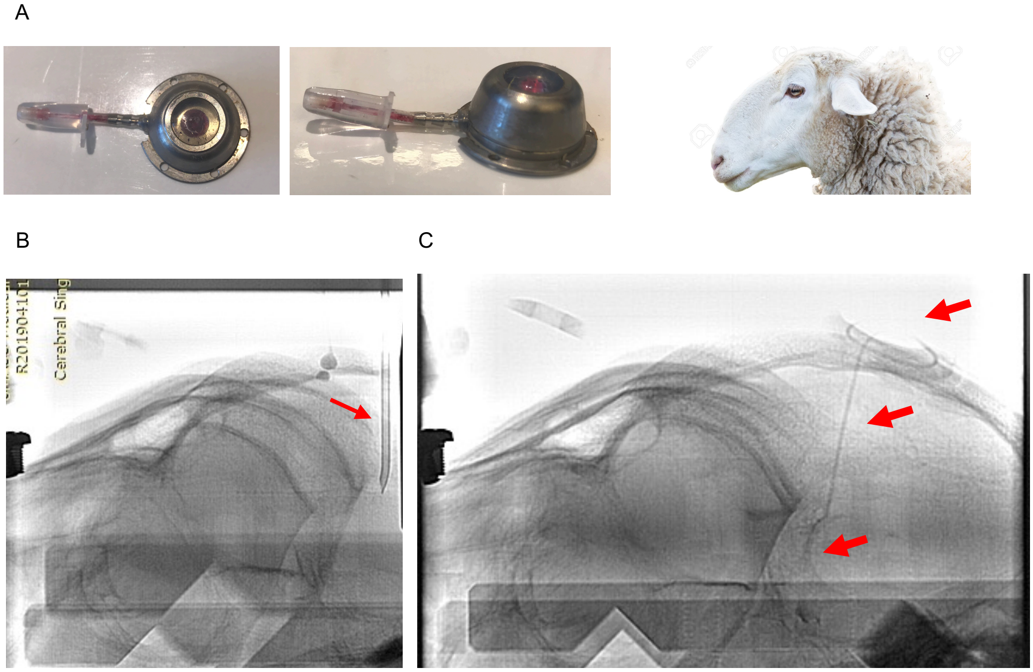

Supplementary Figure 1: Placement of reservoir device into the lateral ventricle of sheep supports repetitive administration of oligonucleotide therapeutics. (a) Reservoir device. (b) CT imaging showing insertion of Tuohy needle into lateral ventricle (red arrow). (c) CT imaging showing catheter into lateral ventricle and distribution of contrast dye into the lateral ventricle.

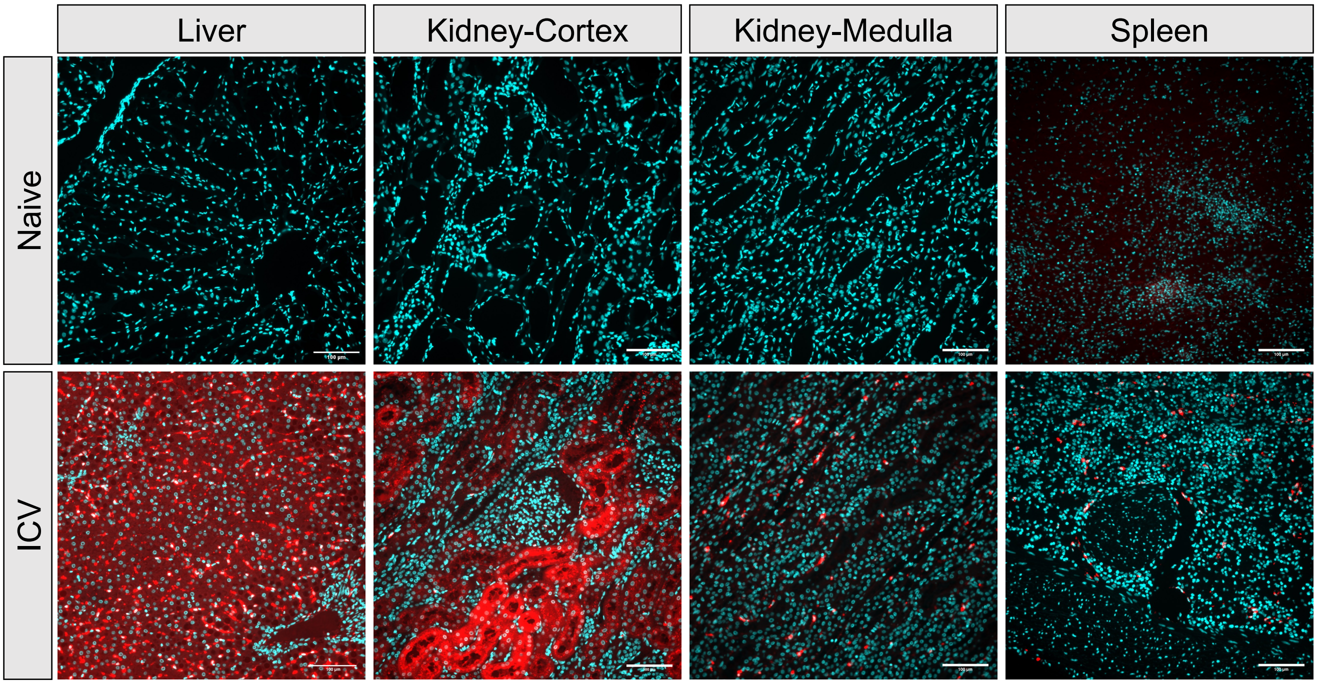

Supplementary Figure 2: Di-siRNA is preferentially cleared by the liver and kidneys. Di-siRNA is primarily cleared in the liver and kidney cortex after administration into the CSF (ICV shown). di-siRNA (cy3, red) in the liver, kidney (cortex and medulla) and spleen after administration into the CSF (ICV shown). Di-siRNA is seen in liver Kupffer cells (left), and kidney epithelial cells (middle). 20x objective, scale bar: 100 µm.

	ICV				ITC			
	Pre		48 hrs		Pre		48 hrs	
	Sheep 1	Sheep 2	Sheep 1	Sheep 2	Sheep 1	Sheep 2	Sheep 1	Sheep 2
ALB	2.6	2.6	2.6	2.7	3.3	3	3.3	2.6
ALP	239	232	177	206	381	709	286	275
ALT	9	24	15	40	17	13	35	763
AMY	12	16	21	17	1	20	22	30
TBIL	0.2	0.3	0.2	0.3	0.2	0.2	0.2	0.3
BUN	10	18	8	8	11	7	12	7
CA	9.6	9.3	10.9	10.1	11.2	11.3	11.1	10
PHOS	9.3	9	10.3	6.6	6.9	8.6	6.8	7
CRE	0.7	0.8	0.6	0.8	0.7	0.8	0.7	0.7
GLU	87	112	81	75	107	105	89	93
NA+	137	139	144	138	142	151	144	152
K+	3.9	5.2	5.2	6.9	5	6.2	5.7	2.9
TP	5.4	5.2	5.8	6.1	6.2	5.9	6.7	5.8
GLOB	2.7	2.7	3.2	3.5	2.9	2.9	3.4	3.2

Supplementary Table 1: Blood chemistry results before (pre) and 48 hours post injection for ICV (left) and ITC (right) administrations. N=2. Alb: albumin; ALP: alkaline phosphatase; ALT: alanine transaminase; AMY: amylase; TBil: total bilirubin; BUN: blood urea nitrogen; CA: Calcium; PHOS: phosphate; CRE: creatinine; GLU: glucose; NA+: sodium; K+: potassium; TP: Total Protein; GLOB: globulin.

	RD Sheep 1								RD Sheep 2							
	Week 1		Week 2		Week 3		Week 4		Week 1		Week 2		Week 3		Week 4	
	Pre-op	48 hrs	Pre-op	48 hrs	Pre-op	48 hrs	Pre-op	48 hrs	Pre-op	48 hrs	Pre-op	48 hrs	Pre-op	48 hrs	Pre-op	48 hrs
aALB	2.9	3	3	3	3	3	2.8	3.2	3	3	2.8	2.9	2.9	3.1	3.2	3.2
ALP	194	167	131	191	217	227	279	295	232	146	127	175	161	214	204	284
ALT	15	46	22	18	18	15	23	19	15	325	58	37	23	23	29	19
AMY	10	12	19	15	19	17	5	15	15	10	16	17	15	18	5	19
TBIL	0.3	0.3	0.3	0.3	0.3	0.3	0.2	0.3	0.4	0.4	0.3	0.3	0.3	0.4		0.3
BUN	15	15	12	12	23	16	8	19	14	11	11	10	16	12	15	16
CA	9.5	10.5	10.5	10.8	10.1	11.2	10	11	9.8	9.8	11.1	11.1	10.2	11.5	11.3	11.3
PHOS	7.9	7.4	8	8.9	7.7	7.4	8.2	8.5	8.9	9.1	8.3	7.5	7.9	8.2	8.2	8.4
CRE	0.3	0.8	0.5	0.7	0.5	0.4	0.5	0.5	0.5	0.6	0.7	0.3	0.7	0.8	0.3	0.7
GLU	123	86	99	83	125	79	197	88	97	52	104	88	164	86	124	79
NA+	138	145	140	140	141	142	139	145	136	141	140	143	138	142	143	146
K+	4	6.9	4.6	5.3	4.8	5	5.1	5.8	4.4	8.5	4.4	4.9	4.4	4.9		5
TP	6	6.9	7.1	6.6	6.5	6.4	6.1	6.5	5.7	7.2	6.7	6.4	6.4	6.5	6.4	6.5
GLOB	3	3.8	4.1	3.6	3.5	3.5	3.3	3.3	2.8	4.2	3.9	3.5	3.5	3.4	3.3	3.3

Supplementary Table 2: Blood chemistry results before (pre) and 48 hours post each RD administrations. N=2. Alb: albumin; ALP: alkaline phosphatase; ALT: alanine transaminase; AMY: amylase; TBil: total bilirubin; BUN: blood urea nitrogen; CA: Calcium; PHOS: phosphate; CRE: creatinine; GLU: glucose; NA+: sodium; K+: potassium; TP: Total Protein; GLOB: globulin.

	RD Sheep 1								RD Sheep 2							
	Week 1		Week 2		Week 3		Week 4		Week 1		Week 2		Week 3		Week 4	
	Pre-op	48 hrs	Pre-op	48 hrs	Pre-op	48 hrs	Pre-op	48 hrs	Pre-op	48 hrs	Pre-op	48 hrs	Pre-op	48 hrs	Pre-op	48 hrs
WBC	10.42	16.12	9.64	9.88	5.81	10.89	10.43	12.25	5.41	10.22	6.78	8.01	5.02	8.42	5.28	9.32
LYM	8.61	10.05	7.41	8.46	4.98	7.74	8.93	9.41	3.84	6.37	5.21	6.16	3.86	6.71	4.67	7.97
MON	0.05	0.08	0.05	0.05	0.03	0.05	0.05	0.06	0.03	0.05	0.03	0.04	0.02	0.04	0.03	0.05
NEU	1.75	5.99	2.18	1.38	0.81	3.1	1.45	2.7	1.54	3.8	1.54	1.81	1.14	1.66	0.58	1.3
RBC	10.09	13.3	11.77	10.87	9.83	11.47	10.35	11.17	9.78	12.58	11.18	10.68	9.49	11.17	9.82	12.77
HGB	10.2	14	12.7	11.1	10.6	12.8	11.5	12.7	10.5	14.2	12.5	11.5	10.1	12.2	10.4	13.9
HCT	25.68	32.02	30.58	28.1	24.46	29.66	27.26	29.61	26.18	34.04	28.33	28.53	25.11	28.69	25.89	33.4
MCV	25	24	26	26	25	26	26	27	27	27	25	27	26	26	26	26
MCH	10.1	10.5	10.8	10.2	10.7	11.1	11.1	11.4	10.7	11.2	11.1	10.7	10.6	10.9	10.6	10.9
PLT	170	222	444	166	226	220	218	233	182	81	538	383	244	411	140	325

Supplementary Table 3: Complete blood counts (CBC) before (pre) and 48 hours post each RD administrations. N=2. WBC: white blood cells; LYM: lymphocytes; MON: monocytes; NEU: neutrophils; RBC: red blood cells; HGB: hemoglobin; HCT: hematocrit; MCV: mean corpuscular volume; MCH: mean corpuscular hemoglobin; PLT: platelets.

**Cortex**			
**Route**	**Mean**	**Std. Deviation**	**Std. Error of Mean**
IS	29.03	0	0
ICV	132.7	31.65	15.83
RD	175	28.65	14.32
ITC	165.2	125.7	62.84
**Caudate**			
**Route**	**Mean**	**Std. Deviation**	**Std. Error of Mean**
IS	950.9	0	0
ICV	65.19	29.16	14.58
RD	174.9	76.07	38.04
ITC	8.967	12.09	6.046
**Putamen**			
**Route**	**Mean**	**Std. Deviation**	**Std. Error of Mean**
IS	1460	0	0
ICV	13.68	10.85	5.423
RD	42.86	27.16	13.58
ITC	5.472	2.774	1.387
**Hippocampus**			
**Route**	**Mean**	**Std. Deviation**	**Std. Error of Mean**
IS	5.816	0	0
ICV	56.41	12.09	6.043
RD	192.6	57.34	28.67
ITC	45.96	12.52	6.262
**Thalamus**			
**Route**	**Mean**	**Std. Deviation**	**Std. Error of Mean**
IS	25.56	0	0
ICV	22.4	10.96	5.478
RD	145.2	73.53	36.76
ITC	87.66	124.9	62.46

Supplementary Table 4: Guide strand accumulation (ug/g) throughout the brain. IS: intrastriatal, ICV: intracerebroventricular, RD: repeated dosing, ITC: intrathecal catheter.

**Cervical**			
**Route**	**Mean**	**Std. Deviation**	**Std. Error of Mean**
IS	6.11	1.693	1.197
ICV	40.22	17.39	8.696
RD	54.57	13.63	6.815
ITC	32.71	9.002	4.501
**Thoracic**			
**Route**	**Mean**	**Std. Deviation**	**Std. Error of Mean**
IS	6.22	1.4	0.9903
ICV	80.85	41.89	20.95
RD	50.48	28.01	14
ITC	42.26	15.23	7.614
**Lumbar**			
**Route**	**Mean**	**Std. Deviation**	**Std. Error of Mean**
IS	3.246	1.117	0.7896
ICV	47.15	25.96	12.98
RD	69.73	15	7.502
ITC	36.5	14.77	7.387

Supplementary Table 5: Guide strand accumulation (ug/g) in the spinal cord between routes of administration. IS: intrastriatal, ICV: intracerebroventricular, RD: repeated dosing, ITC: intrathecal catheter.

CSF			
**Route**	**Mean**	**Std. Deviation**	**Std. Error of Mean**
**ICV**	22.34	1.17	0.8275
**RD**	157	101.2	71.58
**ITC**	87.61	32.76	23.17

Supplementary Table 6: Guide strand accumulation (ng/ul) in the CSF between routes of administration. IS: intrastriatal, ICV: intracerebroventricular, RD: repeated dosing, ITC: intrathecal catheter. One Way ANOVA performed with Graph Pad Prism.

	ICV	RD	ITC
**Cortex**	29.57	19.60	36.96
**Striatum**	0.11	0.14	0.02
**Spinal Cord**	1.68	0.87	1.11
**CSF**	1.12	15.70	4.38
**Total CNS**	32.47	36.31	42.47
**Liver**	32.39	41.73	54.23
**Kidney**	11.39	3.30	5.46
**Spleen**	1.19	0.70	2.46
**Blood**	0.27	0.83	0.17

Supplementary Table 7: Estimate of percent injected dose retained in each tissue after ICV, RD, and ITC siRNA administration.

Liver			
**Route**	**Mean**	**Std. Deviation**	**Std. Error of Mean**
IS	1.64	0	0
ICV	48.2	11.52	8.144
RD	124.2	27.44	19.41
ITC	80.7	9.357	6.617
**Kidney**			
IS	1.103	0	0
ICV	56.95	39.1	27.65
RD	33.03	15.14	10.7
ITC	27.31	6.932	4.902
**Spleen**			
IS	1.04	0	0
ICV	23.88	23.07	16.31
RD	28.16	15.94	11.27
ITC	49.23	58.39	41.29

Supplementary Table 8: Summary table showing guide strand accumulation (ug/g) in the major clearance organs (Liver, Kidney, Spleen) in all routes of administration. IS: intrastriatal, ICV: intracerebroventricular, RD: repeated dosing, ITC: intrathecal catheter.

Cortex					
Dunnett's multiple comparisons test	Mean Diff.	95.00% CI of diff.	Below threshold?	Summary	Adjusted P Value
ICV vs Naïve	70.48	54.38 to 86.57	Yes	****	<0.0001
RD vs Naïve	75.5	59.40 to 91.59	Yes	****	<0.0001
ITC vs Naïve	57.04	40.94 to 73.13	Yes	****	<0.0001
IS vs Naïve	34.13	8.676 to 59.58	Yes	**	0.0089
**Caudate**					
Dunnett's multiple comparisons test	Mean Diff.	95.00% CI of diff.	Below threshold?	Summary	Adjusted P Value
ICV vs Naïve	62.7	33.09 to 92.32	Yes	***	0.0003
RD vs Naïve	51.06	19.07 to 83.05	Yes	**	0.0028
ITC vs Naïve	30.22	0.6060 to 59.84	Yes	*	0.0452
IS vs Naïve	64.98	18.15 to 111.8	Yes	**	0.0075
**Putamen**					
Dunnett's multiple comparisons test	Mean Diff.	95.00% CI of diff.	Below threshold?	Summary	Adjusted P Value
ICV vs Naïve	50.99	0.8916 to 101.1	Yes	*	0.0459
RD vs Naïve	64.39	10.83 to 117.9	Yes	*	0.0192
ITC vs Naïve	2.708	-47.39 to 52.80	No	ns	0.9993
IS vs Naïve	66.71	-9.027 to 142.4	No	ns	0.0877
**Hippocampus**					
Dunnett's multiple comparisons test	Mean Diff.	95.00% CI of diff.	Below threshold?	Summary	Adjusted P Value
ICV vs Naïve	55.1	30.79 to 79.41	Yes	***	0.0001
RD vs Naïve	60.4	36.09 to 84.71	Yes	****	<0.0001
ITC vs Naïve	33.93	9.620 to 58.24	Yes	**	0.0068
IS vs Naïve	11.12	-27.32 to 49.56	No	ns	0.842
**Thalamus**					
Dunnett's multiple comparisons test	Mean Diff.	95.00% CI of diff.	Below threshold?	Summary	Adjusted P Value
ICV vs Naïve	37.33	-1.718 to 76.37	No	ns	0.062
RD vs Naïve	48.43	6.255 to 90.60	Yes	*	0.024

ITC vs Naïve	30.84	-8.204 to 69.88	No	ns	0.1378
IS vs Naïve	30.27	-31.46 to 92.01	No	ns	0.4833

Supplementary Table 9: Statistical comparison of target mRNA knockdown in the brain between routes of administration. IS: intrastriatal, ICV: intracerebroventricular, RD: repeated dosing, ITC: intrathecal catheter. One Way ANOVA performed with Graph Pad Prism.
